# A machine learning-assisted model for renal urate underexcretion with genetic and clinical variables among Chinese men with gout

**DOI:** 10.1186/s13075-022-02755-4

**Published:** 2022-03-09

**Authors:** Mingshu Sun, Wenyan Sun, Xuetong Zhao, Zhiqiang Li, Nicola Dalbeth, Aichang Ji, Yuwei He, Hongzhu Qu, Guangmin Zheng, Lidan Ma, Jiayi Wang, Yongyong Shi, Xiangdong Fang, Haibing Chen, Tony R. Merriman, Changgui Li

**Affiliations:** 1 Shandong Provincial Clinical Research Center for Immune Diseases and Gout & Shandong Provincial Key Laboratory of Metabolic Diseases, The Affiliated Hospital of Qingdao University, Qingdao, China; 2 CAS Key Laboratory of Genome Sciences and Information, Beijing Institute of Genomics, Chinese Academy of Sciences, Beijing, China; 3 Department of Medicine, University of Auckland, Auckland, New Zealand; 4 Department of Pharmacy, Peking University First Hospital, Beijing, China; 5 Department of Endocrinology and Metabolism, Shanghai 10th People’s Hospital, Tongji University, Shanghai, China; 6 Department of Biochemistry, University of Otago, Dunedin, New Zealand; 7 Department of Medicine, University of Alabama Birmingham, Birmingham, AL USA

**Keywords:** Gout, Hyperuricemia, Fractional excretion of uric acid, Single nucleotide polymorphism, Prediction model

## Abstract

**Objectives:**

The objective of this study was to develop and validate a prediction model for renal urate underexcretion (RUE) in male gout patients.

**Methods:**

Men with gout enrolled from multicenter cohorts in China were analyzed as the development and validation data sets. The RUE phenotype was defined as fractional excretion of uric acid (FE_UA_) <5.5%. Candidate genetic and clinical features were screened by the least absolute shrinkage and selection operator (LASSO) with 10-fold cross-validation. Machine learning algorithms (stochastic gradient descent (SGD), logistic regression, support vector machine) were performed to construct a predictive classifier of RUE. Models were assessed by the area under the receiver operating characteristic curve (AUC) and the precision-recall curve (PRC).

**Results:**

One thousand two hundred thirty-eight and two thousand twenty-three patients were enrolled as the development and validation cohorts, with 1220 and 754 randomly chosen patients genotyped, respectively. Rs3775948.GG of *SLC2A9/GLUT9*, rs504915.AA of *NRXN2/URAT1*, and 7 clinical features (age, hypertension, nephrolithiasis, blood glucose, serum urate, urea nitrogen, and creatinine) were generated by LASSO. Two additional SNP variants (rs2231142.GG of *ABCG2* and rs11231463.GG of *SLC22A9/OAT7*) were selected based on their contributions to gout in the development cohort and their reported effects on renal urate handling. The optimized classifiers yielded AUCs of ~0.914 and PRCs of ~0.980 using these 11 variables. The SGD model was conducted in the validation cohort with an AUC of 0.899 and the PRC of 0.957.

**Conclusions:**

A prediction model for RUE composed of four SNPs and readily accessible clinical features was established with acceptable accuracy for men with gout.

**Supplementary Information:**

The online version contains supplementary material available at 10.1186/s13075-022-02755-4.

## Introduction

Gout is the most common inflammatory arthritis with multi-organ involvement, affecting <1% to 6.8% of the general population around the world, and is becoming more prevalent with younger age-at-onset [1–4]. Hyperuricemia is the biochemical basis of gout, with long-term urate-lowering therapy (ULT) a key element of gout management. The pathogenic causes of primary hyperuricemia include urate overproduction in the liver and renal or extra-renal urate underexcretion, depending on the enzymes or urate transporters involved [5, 6].

Fractional excretion of uric acid (FE_UA_) is currently acknowledged as a precise measurement of renal urate clearance, with the normal FE_UA_ range of 5.5–11.1% [7, 8]. Those with FE_UA_ less than 5.5% are classified as with RUE [9]. Accurate assessment of RUE requires assessment of FE_UA_, with 24-h urine sample under a 2-week washout period and 5-day purine-restricted diet considered the standard measurement [10], although there are studies exploring spot or a few hour urine samples as the substitutions [7–9, 11]. Given the fact that many drugs may interfere with renal urate excretion, all patients were required to withdraw all drugs during the washout time. However, withdrawal of medications during the washout period can be problematic. In addition, the inconvenience of 24-h urine collection and strict life control limits its application in daily practice. A simple and reliable method to identify patients with RUE is needed, both for research purposes and also in clinical practice, particularly when assessing younger patients with gout and those with a strong family history of gout.

Genetic and clinical research based on big data has set the stage for the development of prediction models with genetic and/or clinical variables in recent years. Genome-wide association studies (GWAS) have revealed urate-associated genetic variants, some of which are within genes of urate transporters or their regulators [12, 13]. Other studies have also reported genotypes associated with urate export parameters or even renal urate handling profiles [14, 15]. Many of the candidate loci and variants are causally associated with serum urate concentration, for example, those in *SLC2A9/GLUT9*, *ABCG2*, and *SLC22A12/URAT1*, and some are rather marker SNPs which are in linkage disequilibrium with a candidate causal SNP, like rs1797052T of *PDZK1* [12, 16]*.* In a large participating general population-based pedigree study, 183 index SNPs identified in a trans-ancestry GWAS for serum urate levels explained 17% of heritability [5]. Additionally, a number of clinical variables including body mass index (BMI), age, and renal function are associated with renal urate handling [15]. These data provide the possibility to investigate prediction models for gout pathogenic phenotypes using genetic or readily accessible clinical data.

So far, no such models are available. This study was designed to investigate a prediction model for RUE in men with gout. First, RUE, defined as FE_UA_ <5.5%, was measured in a gout cohort from a single center in China, and gout and/or hyperuricemia-associated SNPs identified to be East Asian-specific as previously reported were genotyped [12, 13, 16]. Then we developed machine learning (ML) prediction models for clustering the RUE phenotype using genetic and clinical variables. The models were validated in a multicenter cohort from three Chinese hospitals.

## Methods

### Study population

Male patients with gout that met the 2015 American College of Rheumatology/European League against Rheumatism classification criteria were enrolled [17]. Exclusion criteria included blood pressure ≥180/110 mmHg, blood glucose ≥11.1 mmol/L, eGFR <45 ml/min/1.73m^2^, taking regular anticoagulant and with severe heart, kidney, or brain disease; cancer; or mental or metabolism disorders. For each subject enrolled, 24-h urine sample was collected after a 14-day washout period of any drug and low purine diet (purine intake <200 mg/day) for 5 days [10].

**Fig. 1:**
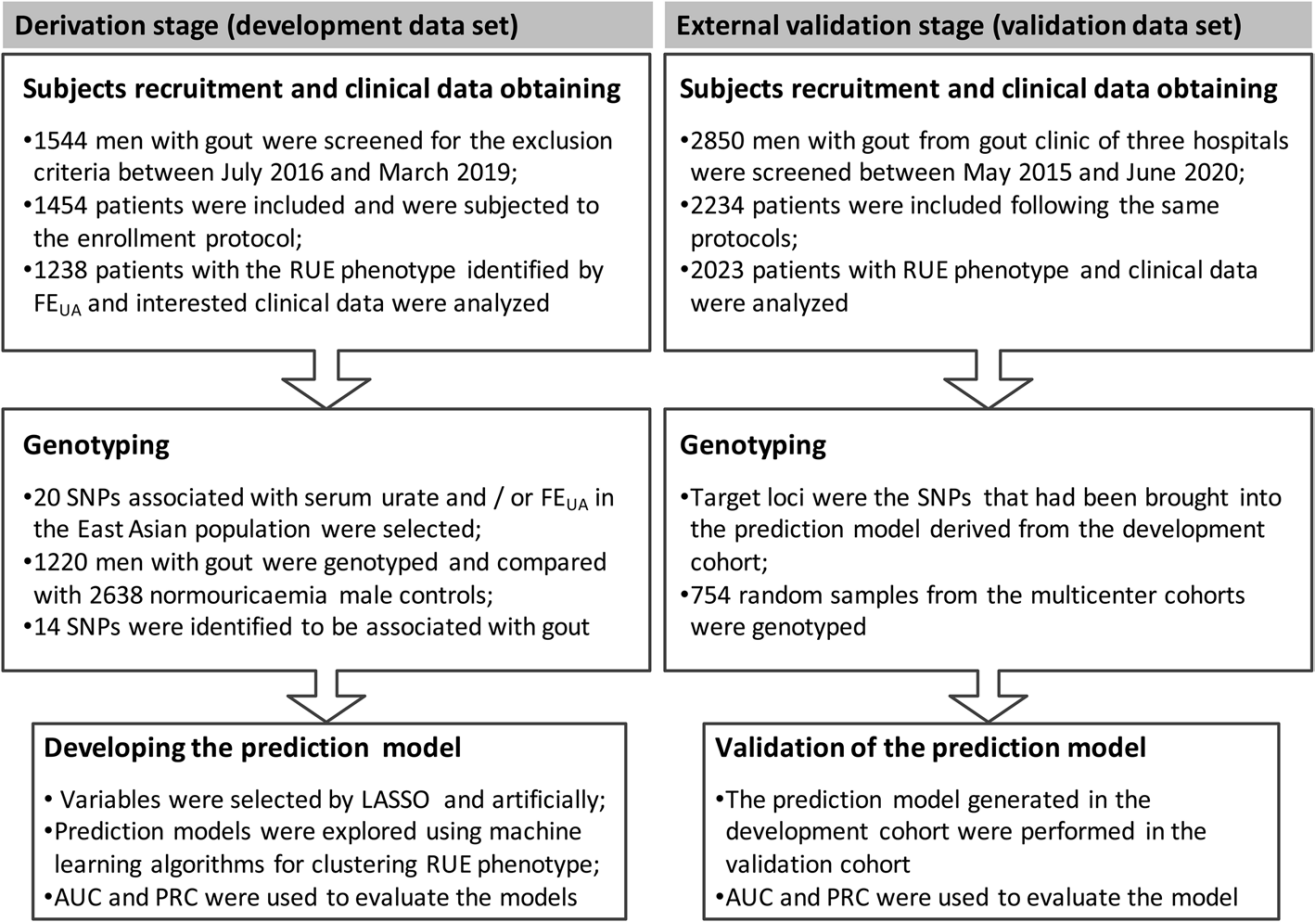
Flow chart of the study. RUE, renal urate underexcretion; FE_UA_, fractional excretion of uric acid; SNP, single nucleotide polymorphism; LASSO, least absolute shrinkage and selection operator; AUC, area under the receiver operating curve; PRC, precision-recall curve

Men who attended the Shandong Provincial Gout Clinical Medical Center, the Affiliated Hospital of Qingdao University (Qingdao, China) between July 2016 and March 2019 comprised the development data set. Men from gout clinics of Shanghai Jiaotong University Affiliated 6th People’s Hospital (Shanghai, China), Tongji University Affiliated 10th People’s Hospital (Shanghai, China), and the Affiliated Hospital of Qingdao University (Qingdao, China) between May 2015 and June 2020 served as the validation data set. The overall study design is shown in Fig. 1.

This study was approved by the Ethics Committee of the Affiliated Hospital of Qingdao University (Qingdao, China). All participants gave their written informed consents.

### Clinical variables, detection of RUE subtype, and statistical analysis

Clinical data were obtained from each hospital’s electronic health record system, including demographic and medical information, height, weight, waistline, systolic blood pressure (SBP), diastolic blood pressure (DBP), tophi, and biochemical parameters. Serum urate (SU), blood glucose (Glu), triglyceride (TG), total cholesterol (TC), low-density lipoprotein (LDL), high-density lipoprotein (HDL), blood urea nitrogen (BUN), serum creatinine (sCr), urinary uric acid (uUA), and urinary creatinine (uCr) were detected using an automatic biochemical analyzer (TBA-40FR; TOSHIBA, Japan).

Parameters for renal urate handling were measured by FE_UA_, which was the percentage of renal urate clearance over creatinine clearance (FE_UA_ = uUA/uCr × sCr/SU). Participants with FE_UA_<5.5% were defined as with RUE [9].

The characteristics of the overall study patients are described in Table 1. For continuous covariates, summary statistics are reported as mean (standard deviation) or median (interquartile range), where appropriate. Proportions were compared using the Chi-square test and continuous variables were compared using ANOVA or Kruskal-Wallis tests, as appropriate. Univariate and multiple linear regression analyses were performed to investigate the effect of clinical features on FE_UA_ in the pooled gout patients [18, 19]. SPSS 25.0 software was used for all analyses. A two-sided *p* < 0.05 was designated as statistically significant for all analyses.

**Table 1:** Comparison of clinical features among development and validation data sets

**Parameters**	**Development**	**Validation**
Number	1238	2023
Age, years	42 (32–53)	43 (33–57)^**^
BMI, kg/m^2^	27.0 (24.8–29.3)	26.0 (24.1–28.4)^**^
SBP, mmHg	129 (120–140)	130 (120–140)
DBP, mmHg	81 (75–90)	82 (77–90)^**^
SU, μmol/L	522 (462–585)	522 (455–589)
Glu, mmol/L	5.38 (5.00–5.77)	5.46 (5.13–5.90)^**^
TG, mmol/L	1.73 (1.22–2.50)	1.99 (1.39–2.88)^**^
TC, mmol/L	4.80 (4.26–5.44)	4.89 (4.30–5.52)
LDL-C, mmol/L	3.46 (2.88–4.04)	3.06 (2.52–3.61)^**^
HDL-C, mmol/L	1.05 (0.89–1.22)	1.02 (0.89–1.19)
BUN, mmol/L	4.29 (3.60–5.10)	4.60 (3.90–5.40)^**^
sCr, μmol/L	81.00 (73.00–90.00)	85.00 (76.00–95.00)^**^
eGFR, ml/min/1.73 m^2^	96.3 (84.9–110.1)	90.9 (78.5–105.7)^**^
Tophi, *n* (%)	380 (37.1)	-
Nephrolithiasis, *n* (%)	173 (17.9)	715 (35.3)^**^
Hypertension, *n* (%)	215 (21.5)	142 (7.0)^**^
Cardiovascular disease, *n* (%)	20 (2.0)	36 (1.8)
Diabetes mellitus, *n* (%)	24 (2.4)	30 (1.5)
Smoking, *n* (%)	355 (35.6)	705 (35.1)
Drinking, *n* (%)	749 (74.9)	1022 (50.7)^**^
Family history^a^, *n* (%)	503 (49.2)	-
FE_UA_, %	4.2 (3.4–5.0)	4.0 (3.3–4.9)^*^
Adjusted FE_UA_^b^, %	4.21 (0.99)	4.26 (1.37)
RUE, *n* (%)	83.4%	83.0%

Parameters are displayed with mean (standard deviation) or median (interquartile range)

*BMI* Body mass index, *SBP* Systemic blood pressure, *DBP* Diastolic blood pressure, *SU* Serum urate, *Glu* Fasting blood glucose, *TG* Triglyceride, *TC* Total cholesterol, *LDL-C* Low-density lipoprotein-cholesterol, *HDL-C* High-density lipoprotein-cholesterol, *BUN* Blood urea nitrogen, *sCr* Serum creatinine, *eGFR* Estimated glomerular filtration rate, *FE*_*UA*_ Fractional excretion of urinary urate, *UUE* 24-h urinary urate amount

^*^Compared with the development data set, *p*<0.05; ^**^compared with the development data set, *p*<0.001

^a^Family history of gout, hyperuricemia, diabetes mellitus, hypertension or cardiovascular disease

^b^Adjusted for age, BMI, SU, Glu, TG, BUN, sCr, nephrolithiasis, hypertension, cardiovascular disease, and tophi; -, data missing

### Genotyping and statistical analysis

The target genetic variations were 20 single nucleotide polymorphisms (SNPs) identified as gout-risk loci or associated with SU concentrations and FE_UA_ in the East Asian population as previously reported [12, 13]. Genomic DNA was extracted from peripheral blood mononuclear cells. Genotyping of the selected SNPs were performed with a SpectroCHIP®II-G384 array (Agena Bioscience, San Diego, USA). In the development data set, all 20 target SNPs were tested. 2638 controls were from the Chinese healthy male sample set of our previous gout GWAS to identify candidate SNPs associated with gout for the purposes of modeling [20]. Only SNPs included in the prediction model were tested in the validation data set. Association analyses of SNPs with the FE_UA_ were done using PLINK (http://www.bwh.harvard.edu/plink/) and association analyses of these loci with clinical variables were done using an additive genetic model implemented in SNPTEST (http://mathgen.stats.ox.ac.uk/genetics_software/snptest/snptest.html) (A two-sided *p*<0.0025 was assumed to be significant for the SNP association analyses). Association of the *z*-score of the residuals with SNP allele dose was tested by linear regression.

### Prediction model analysis

Men with gout in the development data set with complete clinical and genetic data of interest were included for variable selection and classifier construction. Patients were classified with or without the RUE phenotype according to FE_UA_ <5.5% versus FE_UA_ ≥5.5%, respectively. Samples of the development data set were randomly divided into training and test sets (5:1). As described herein, 31 clinical and biochemical variables, as well as candidate SNP information were screened by Least Absolute Shrinkage and Selection Operator (LASSO) regression [21], augmented with 10-fold cross-validation in the training set for internal validation. Imputation for missing variables was performed if missing values were no more than 20%. The most predictive covariates to RUE phenotype were selected by the minimum criteria (lambda.min). The R package “glmnet” statistical software (R Foundation) was used to perform the LASSO regression. Subsequently, variables identified by LASSO regression analysis were entered into ML models to construct a classifier to identify RUE phenotype. We used three ML algorithms to perform modeling by a Python script, which were stochastic gradient descent (SGD) [22], logistic regression (LG), and linear support vector classifier (SVC). An external multicenter validation set of gout cases with complete data was employed to validate the classifier performance. The area under the receiver operating curve (AUC) and the precision-recall curve (PRC) were used to evaluate the prediction efficacy of the models. R software (http://www.R-project.org) and Python were used for all modeling analyses.

Another filter, the classical extreme gradient boosting (XGBoost) method, and other classifiers, random forests and neural networks, were also performed for comparisons to LASSO and the modeling algorisms described above.

## Results

### Clinical characteristics of the two data sets

A total of 1238 and 2023 male patients with gout from three hospitals were included and analyzed as the development and validation cohorts, respectively (Table 1). We also explored the effect of clinical variables on FE_UA_ in the pooled group of the two cohorts by linear regression analyses (Table 2). Overall, FE_UA_ was comparable between the two sets (4.21% vs 4.26%, *p*>0.05), after adjusting for age, BMI, other biochemical parameters, and the presence of nephrolithiasis, cardiovascular disease, hypertension, and tophi that were associated with FE_UA_ in the univariate regression analysis. The proportion of patients with RUE was also comparable between the two data sets (83.4% vs 83.0%, *p*>0.05). Multiple linear regression models for FE_UA_ showed that age, Glu, BUN, sCr, and the presence of hypertension or nephrolithiasis were independent positive predictors (*p*<0.05), while SU was a negative predictor for FE_UA_ (*p*<0.05).

**Table 2:** Linear regression analyses of clinical variables with FE_UA_ (%) in the pooled group of gout patients

	**Univariate** ***β*** **(s.e.)**	**Multivarite** ***β*** **(s.e.)**
Age, per 10 years	0.359 (0.021)^**^	0.119 (0.039)^*^
BMI, kg/m^2^	− 0.032 (0.009)^**^	0.007 (0.009)
SU, per 60 μmol/L	− 0.471 (0.015)^**^	− 0.321 (0.023)^**^
Glu, mmol/L	0.324 (0.031)^**^	0.267 (0.040)^**^
TG, mmol/L	− 0.040 (0.017)^*^	− 0.003 (0.022)
TC, mmol/L	0.004 (0.033)	
BUN, mmol/L	0.286 (0.022)^**^	0.203 (0.029)^**^
sCr, per 10 μmol/L	0.170 (0.020)^**^	0.320 (0.078)^**^
Tophi	0.246 (0.080)^*^	0.145 (0.071) ^*^
Nephrolithiasis	0.153 (0.069)^*^	0.279 (0.084)^*^
Hypertension	0.374 (0.095)^**^	0.222 (0.075)^*^
Cardiovascular disease	0.547 (0.251)^*^	− 0.026 (0.218)
History of smoking	0.053 (0.065)	
History of drinking	0.030 (0.063)	
Family history	0.016 (0.079)	

*s.e.* Standard error, *FE*_*UA*_ Fractional excretion of urinary urate, *BMI* Body mass index, *SU* Serum urate, *Glu* Fasting blood glucose, *TG* Triglyceride, *TC* Total cholesterol, *HDL-C* High-density lipoprotein-cholesterol, *BUN* Blood urea nitrogen, *sCr* Serum creatinine

^*^p<0.05; ^**^p<0.001

### Association of SNPs with gout, SU levels, and FE_UA_ in the development data set

1220 patients in the development set were genotyped for the 20 SNPs. Compared to 2638 urate-normal non-gout controls based on association analysis, 42 variants of 14 SNPs were identified as gout-associated and served as candidate genetic variables for modeling, which were *ABCG2*, *MUC1*, *PDZK1*, *GCKR*, *SLC2A9/GLUT9* (2 loci), *SLC22A9/OAT7*, *PLA2G16*, *FLRT1*, *NRXN2/URAT1* (2 loci), *AIP*, *ALDH2*, and *COMMD4*. The other 6 SNPs were rs2762353 of *SLC17A1*, rs17145750 of *MLXIPL*, rs79105258 of *CUX2*, rs4966024 of *IGF1R*, rs73575095 of *MAF*, and rs9895661 of *BCAS3*, which are mostly associated with metabolic pathways or inflammation despite *SLC17A1* and are not biologically immediately linked to renal urate handling. Odds ratio and 95% confidence intervals of each SNP allele are shown in Supplementary Table S1.

We also evaluated the association of the 14 candidate SNPs with SU levels and FE_UA_ (Table 3) in the development data set. Only rs2231142 of *ABCG2* showed nominal association (*β*=0.155, *p*<2.5×10^−3^) with SU level. SNPs at three loci showed nominal association with FE_UA_, which were one at *ABCG2* and two at *SLC2A9*.

**Table 3:** Association analyses between 14 candidate SNPs and serum urate and FE_UA_ in the development cohort

**Gene**	**SNP**	**A1**	**A2**	**Serum urate (μmol/L)**			**FE**_**UA**_ **(%)**		
				**Effect**	**s.e.**	***p *value**	**Effect**	**s.e.**	***p *value**
PDZK1	rs1797052	T	C	− 0.082	0.048	0.090	0.017	0.048	0.720
MUC1	rs4072037	C	T	− 0.049	0.073	0.501	0.129	0.073	0.076
GCKR	rs1260326	C	T	0.089	0.041	0.031	− 0.114	0.041	0.006
SLC2A9	rs7679724	G	T	0.063	0.044	0.155	− 0.206	0.044	3.05E−6^*^
SLC2A9	rs3775948	G	C	− 0.020	0.065	0.758	0.208	0.065	0.001^*^
ABCG2	rs2231142	T	G	0.155	0.040	1.03E−4^*^	0.154	0.040	1.07E−4^*^
SLC22A9	rs11231463	G	A	− 0.061	0.063	0.332	− 0.114	0.063	0.068
PLA2G16	rs7928514	A	G	− 0.101	0.058	0.080	− 0.055	0.058	0.341
FLRT1	rs641811	A	G	0.020	0.047	0.662	0.001	0.047	0.991
NRXN2	rs57633992	A	C	− 0.061	0.079	0.444	− 0.050	0.079	0.528
NRXN2	rs504915	A	T	− 0.032	0.049	0.516	0.015	0.049	0.756
AIP	rs11227805	T	C	− 0.017	0.066	0.798	0.040	0.066	0.542
ALDH2	rs671	A	G	− 0.017	0.063	0.788	− 0.005	0.063	0.932
COMMD4	rs73436803	T	C	0.339	0.179	0.057	− 0.193	0.178	0.279

*A1* Allele 1, effect allele, *s.e.* Standard error, *FE*_*UA*_ Fractional excretion of uric acid

^*^p<0.0025 as significant

### Prediction model

**Fig. 2:**
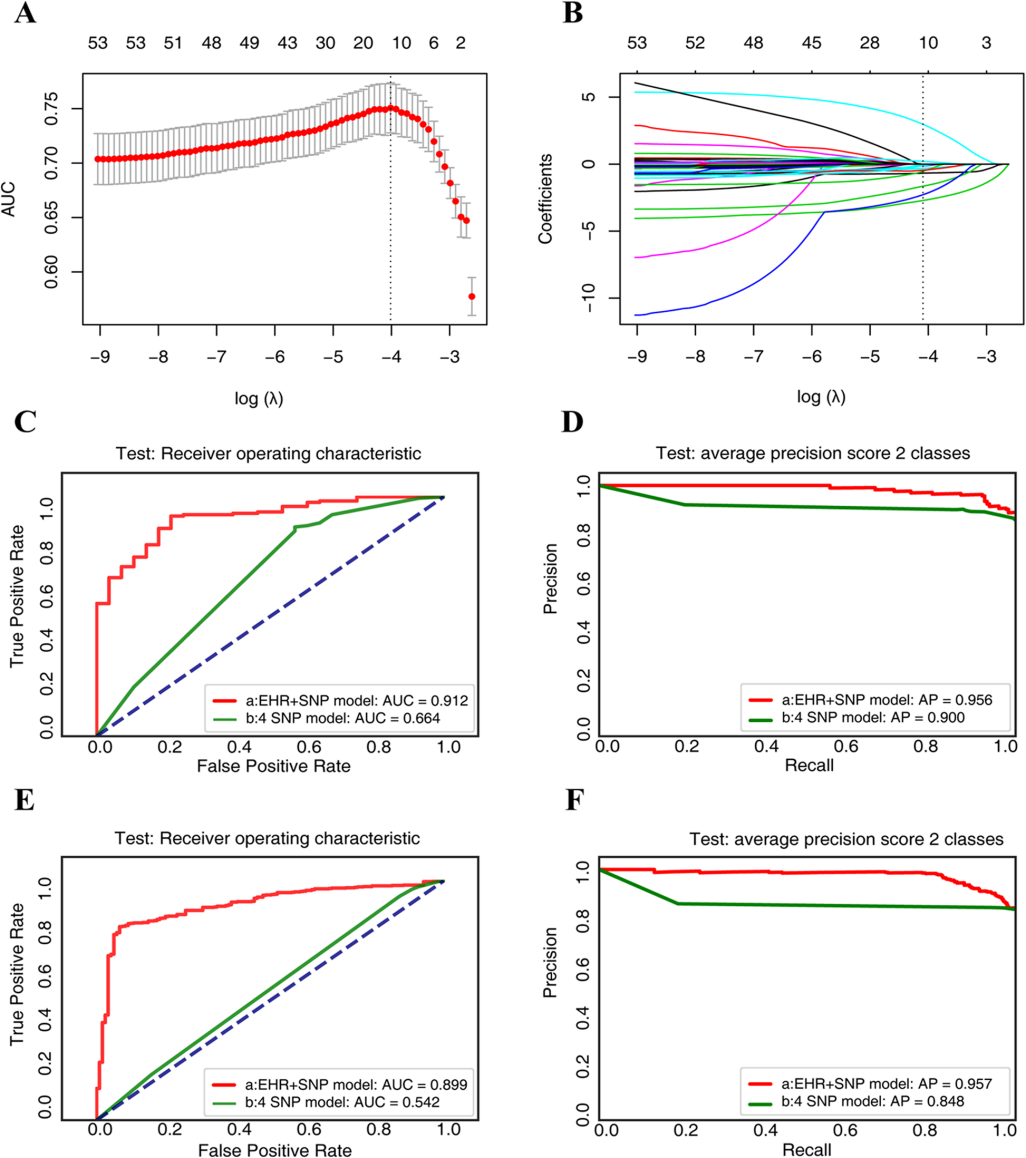
**Prediction modeling of gout patients with urate renal underexcretion (RUE).** **A** The area under the receiver-operator characteristic curve (AUC) of different numbers of 73 variables (42 SNP variations and 31 clinical parameters) revealed by the LASSO model in the derivation set. The red dots represent the AUC score, the gray lines represent the standard error, and the vertical dotted lines represent optimal values by minimum criteria. The upper abscissa is the number of non-zero coefficients in the model at this time, the lower abscissa is log λ, which is the tuning parameter used for 10-fold cross-validation in the LASSO model. A dotted vertical line is drawn at the optimal values by minimum criteria, which is 11. **B** LASSO coefficient profiles of the 73 variables. A vertical line is drawn at the optimal value by 1−SE criteria and results in 11 non-zero coefficients. **C** The receiver-operator characteristic analyses for predicting RUE in the internal test set with stochastic gradient descent. **D** The precision-recall curve of predicting RUE in the internal test set. **E** The receiver-operator characteristic analyses for predicting RUE in the validation set with stochastic gradient descent. **F** The precision-recall curve of predicting RUE in the validation set

By applying the LASSO algorithm to the 42 genetic variants and 31 clinical variables in the training sample, the important variables for identifying RUE were determined, with the log (λ) values being summarized in Fig. 2A and B. Four SNP variations (rs7679724.TT and rs3775948.GG of *SLC2A9/GLUT9*, rs504915.AA of *NRXN2/URAT1*, and rs11227805.TT of *AIP*) and 7 clinical features (age, hypertension, nephrolithiasis, Glu, SU, BUN, and sCr) were selected by LASSO as the most important for predicting RUE phenotype. The ML models (Linear SVC, SGD, and LG) predicted the RUE with AUCs of ~0.622 using the 4 SNP variables, and ~0.899 using the combo of the 11 genetic and clinical variables in the internal test set (Supplementary Table S2).

To enhance the prediction efficacy of genetic predictors, we selected two additional variations, rs2231142.GG of *ABCG2* and rs11231463.GG of *SLC22A9/OAT7*, based on their contributions to gout in the development cohort (Supplementary Table S1) and their effects on renal urate handling as reported [7–9, 11]. We optimized the models by performing with different combinations of these 13 variables following the principle of prediction efficacy and economy. Combining the two additional variations with rs3775948.GG and rs504915.AA, the models yielded higher AUCs of ~0.667, and AUCs of ~0.914 using the combination of the 4 artificially selected genetic variations and the 7 clinical variables (Supplementary Table S2). The SGD model for classifying RUE showed an AUC of 0.912 (95% CI 0.894 to 0.920) and a PRC of 0.956 in the internal test sets (Fig. 2C, D). The prediction performance of the SGD model in the external validation cohort (*n*=754) yielded an AUC of 0.899 (95% CI 0.887 to 0.904) and a PRC of 0.956 (Fig. 2E, F).

The RUE phenotype risk score was constructed based on the coefficients from the SGD model. The probability was calculated as following: *f*(*x*) = 1/[1 + *e* ( − *x*)], which was the mean after tenfold cross-validations. A calculator of the ML SGD model was developed to allow local clinicians to enter the values of the 4 SNP variations and 7 clinical variables required for the risk score with automatic calculation of the likelihood that a gout patient is a renal underexcretor.

The XGBoost obtained 18 features, which were SU, GLU, eGFR, Ccr, BUN, BMI, rs3775948.GG, LDL, DBP, sCr, age, nephrolithiasis, rs7679724.TT, history of smoking, SBP, hypertension, rs57633992.AC, and rs2762353.GG. The models developed using XGBoost selected variables, random forests or neural networks achieved the AUCs of 0.864~0.904. The results were presented in Supplementary Tables S2 and S3.

## Discussion

Prediction models for individual disease diagnosis, incidence, or outcome are growing rapidly in recent years, with the development of new learning algorithms and the ongoing explosion of data. Good prediction models provide useful tools in disease management and greatly ease the clinical practice [23]. Here we developed for the first time a ML prediction model for the risk of RUE phenotype in gout patients. Eleven variables were selected by the LASSO algorithm or artificial selection based on their importance in modeling or impacts on SU and FE_UA_, respectively. We established 3 ML prediction models for RUE using reliable genetic variants and easily accessible clinical features with stable and acceptable efficacy (AUC=0.91) and validated the model in a multicenter gout cohort. By comparing with XGBoost filter and other classifiers, the neural network Multi-layer Perceptron Classifier, and Random Forest, we confirmed that the models displayed here are the optimal ones with the highest predicting accuracy. A calculator based on the ML SGD model using these predictors was generated and readily available in the clinic, enabling clinicians to estimate the probability of a patient with RUE.

Four SNPs were selected by LASSO as the most important contributors for grouping RUE. However, the prediction accuracy was only about 0.62 in the ML models using these 4 genetic variables. We tried an artificial selection of the SNP variants to improve the prediction efficacy of the model. The rs2231142.T allele causes dysfunction of ABCG2, a urate transporter mainly located in the intestinal tract, and was previously demonstrated to be associated with extra-renal underexcretion [9]. It was the only locus with nominal association with SU level in our development data set and was significantly associated with FE_UA_. The rs11231463.G allele of *SLC22A9* increased the risk of gout by 2.2 times compared with urate-normal controls in this study. *SLC22A9* encodes OAT7, which is expressed in the liver and exhibits modest uricosuric-sensitive urate uptake activity [12, 13]. These two variants were also selected according to their effectiveness on SU and FE_UA_. Two SNPs selected by LASSO, rs3775838.GG of *SLC2A9* and rs504915.AA of *NRXN2/SLC22A12*, were adopted in the model. *SLC2A9* encodes GLUT9, and *NRXN2/SLC22A12* encodes URAT1, both of which were the major urate exporters located in renal tubules. These two variants were both associated with gout in the development cohort. By combining these two LASSO selected genetic variants with the two additionally selected variants, and also with the clinical variables, the prediction capacity of the ML models was optimized (Supplementary Table S2).

Additional clinical variables were ranked and the top 7 were selected by LASSO, including 3 medical history features (age, presence of hypertension, and nephrolithiasis) which are easy to obtain, and 4 biochemical parameters (Glu, SU, BUN, and sCr). These predictors included by ML algorithm are statistically significant contributors to FE_UA_ in the pooled gout group and are also biologically meaningful. Aging and elevated sCr indicate impaired kidney function. In the early stage of kidney dysfunction (in this study, eGFR ≥45 ml/min/1.73m^2^), the tubular reabsorption function is the dominant problem contributing to renal urate handling, which may manifest as increased FE_UA_. It is consistent with earlier research, in which urate reabsorption in the tubular was reduced, accompanied by increased urate excretion during the early stage of gouty nephropathy [24]. BUN shares a similar renal tubular excretion pattern with urate, but may compete with urinary urate during the process of tubule reabsorption and secretion. Since there is an osmotic diuretic effect of urinary glucose, it is reasonable to predict that FE_UA_ would increase with blood glucose. Hypertension may increase the glomerular filtration and induce a hyperfiltration-associated urinary urate excretion [25]. A high level of SU is a burden to renal tubule secretion rather than glomerular filtration [26]. Healthy subjects have increased FE_UA_ corresponding to SU elevation while most gout patients do not, implying that intrinsic defects may exist in renal tubule urate handling of these patients [7, 10]. At last, the presence of nephrolithiasis may be an indicator for renal urate excretion. Combining these 7 clinical features with the SNP variable combination, three ML models were performed and produced similar AUCs of approximately 0.9 in the development and multicenter validation cohorts.

To ensure the reliability and effectiveness of the model, multicenter cohorts of Chinese male adults with gout were adopted to serve as the development and the external validation data set in this study. The distributions of patients with RUE (83.4% versus 83%) are strictly consistent between analyses conducted in the two data sets, which also fits the same profile as existing reports [27]. Besides, vital clinical characteristics, the SU and FE_UA_ levels, are also identical between them. These features of the two data sets promised an appropriate data environment for the prediction model development and the reliability of any model generated.

Furthermore, the RUE phenotype was profiled using a standard method. RUE used to be defined solely based on the absolute 24-h renal urate excretion with the premise that a fixed fraction of daily urate production was excreted by the kidneys [28]. However, the urinary urate amount is a synergistic result of the renal urate load, the glomerular filtration function and the excretion capacity of renal tubules [7, 29–31]. FE_UA_ is a more precise measurement for the identification of low renal uric acid clearance phenotype, which has been adjusted for the SU level and normalized to the individual’s glomerular filtration rate [8].

This study has certain limitations. First is that the efficacy of the SNP predictors in this model is not powerful enough, mostly because that all index SNPs at 183 loci identified by trans-ancestry GWAS can explain only 7.7% of the SU genetic heritability in the East Asian population [5]. The prediction model will be improved by the inclusion of more genetic variants from future genetic research. The second limitation is that this model was established among Chinese gout patients. It should be tested carefully before application in diverse ethnics. The third one is that this model is tested in men alone, and should be validated in female gout cohorts. Finally, despite our efforts to make the prediction model as stable and robust as possible, some variable biochemical parameters like glucose, SU, and BUN were still included, which may have increased the instability of the model. However, these parameters are generally stable when obtained under controlled conditions as described in this study.

## Conclusion

In conclusion, this research developed and validated a reliable and practical model to predict the RUE phenotype in Chinese men with gout, which were helpful for individualized therapy. Additional testing and independent validation would be of benefit, and we provide a calculator to assist with determination in other cohorts.

## Supplementary Information

**Additional file 1: Supplementary Table S1.** Association analyses between 20 SNPs and gout in the development cohort. **Supplementary Table S2.** Performances of different models for RUE in the internal test sets and validation cohort. **Supplementary Table S3.** Performances of models under XGBoost for RUE in the internal test sets.

## Data Availability

The datasets used and analyzed during the current study are available from the corresponding author on reasonable request.

## Abbreviations

RUE: Renal urate underexcretion
FE_UA_: Fractional excretion of uric acid
LASSO: Least absolute shrinkage and selection operator
SGD: Stochastic gradient descent
AUC: The area under the receiver operating characteristic curve
PRC: Precision-recall curve
ULT: Urate-lowering therapy
GWAS: Genome-wide association studies
ML: Machine learning
SBP: Systolic blood pressure
DBP: Diastolic blood pressure
SU: Serum urate
Glu: Blood glucose
TG: Triglyceride
TC: Total cholesterol
LDL: Low-density lipoprotein
HDL: High-density lipoprotein
BUN: Blood urea nitrogen
sCr: Serum creatinine
uUA: Urinary uric acid
uCr: Urinary creatinine
SNPs: Single nucleotide polymorphisms
LG: Logistic regression
SVC: Linear support vector classifier
