# Supplementary material for: A machine learning-assisted model for renal urate underexcretion with genetic and clinical variables among Chinese men with gout
Source: Arthritis Res Ther. 2022 Mar 9;24:67. doi: 10.1186/s13075-022-02755-4 (PMC8905745; doi:10.1186/s13075-022-02755-4)
Supplement: Supplementary file 1 — Additional file 1: Supplementary Table S1. Association analyses between 20 SNPs and gout in the development cohort. Supplementary Table S2. Performances of different models for RUE in the internal test sets and validation cohort. Supplementary Table S3. Performances of models under XGBoost for RUE in the internal test sets. [file 13075_2022_2755_MOESM1_ESM.docx]

**Supplementary Table S1 Association analyses between 20 SNPs and gout in the development cohort**

| **Gene** | **SNP** | **A1** | **A2** | **Gout vs controls** | |
| --- | --- | --- | --- | --- | --- |
|  |  |  |  | **OR** | **95% CI** |
| PDZK1 | rs1797052 | T | C | 1.173 | 1.045-1.316 |
| MUC1 | rs4072037 | C | T | 0.442 | 0.371-0.527 |
| GCKR | rs1260326 | C | T | 0.853 | 0.775-0.940 |
| SLC2A9 | rs7679724 | G | T | 0.670 | 0.606-0.742 |
| SLC2A9 | rs3775948 | G | C | 1.561 | 1.417-1.720 |
| ABCG2 | rs2231142 | T | G | 2.425 | 2.199-2.676 |
| SLC17A1 | rs2762353 | A | G | 0.994 | 0.881-1.121 |
| MLXIPL | rs17145750 | T | C | 0.861 | 0.741-1.001 |
| SLC22A9 | rs11231463 | G | A | 2.169 | 1.771-2.656 |
| PLA2G16 | rs7928514 | A | G | 2.235 | 1.871-2.671 |
| FLRT1 | rs641811 | A | G | 1.750 | 1.522-2.012 |
| NRXN2 | rs57633992 | A | C | 1.823 | 1.469-2.261 |
| NRXN2 | rs504915 | A | T | 0.827 | 0.739-0.925 |
| AIP | rs11227805 | T | C | 1.440 | 1.205-1.720 |
| CUX2 | rs79105258 | A | C | 1.112 | 0.977-1.266 |
| ALDH2 | rs671 | A | G | 0.731 | 0.635-0.842 |
| COMMD4 | rs73436803 | T | C | 0.431 | 0.293-0.633 |
| IGF1R | rs4966024 | A | G | 0.956 | 0.868-1.053 |
| MAF | rs73575095 | C | T | 0.978 | 0.869-1.101 |
| BCAS3 | rs9895661 | C | T | 0.953 | 0.864-1.050 |

A1, allele 1, effect allele; OR, odds ratio; 95% CI, 95% confidence intervals; s.e., standard error; FE_UA_, fractional excretion of uric acid. ^*^, p<0.0025 as significant

**Supplementary Table S2 Performances of different models for RUE in the internal test sets and validation cohort**

|  | AUC (95%CI) | Sensitivity | Specificity | Accuracy | PRC |
| --- | --- | --- | --- | --- | --- |
| Test sets |  |  |  |  |  |
| SGD model |  |  |  |  |  |
| 4SNPs by LASSO + 7Clinical | 0.897 (0.893-0.900) | 0.834 | 0.857 | 0.837 | 0.973 |
| 4SNPs by LASSO | 0.621 (0.608-0.634) | 0.857 | 0.357 | 0.788 | 0.893 |
| LG model |  |  |  |  |  |
| 4SNPs by LASSO + 7Clinical | 0.898 (0.894-0.902) | 0.834 | 0.857 | 0.837 | 0.973 |
| 4SNPs by LASSO | 0.616 (0.595-0.637) | 0.960 | 0.250 | 0.862 | 0.889 |
| SVC model |  |  |  |  |  |
| 4SNPs by LASSO + 7Clinical | 0.899 (0.897-0.902) | 0.840 | 0.857 | 0.842 | 0.974 |
| 4SNPs by LASSO | 0.622 (0.604-0.640) | 0.674 | 0.571 | 0.660 | 0.908 |
| SGD model |  |  |  |  |  |
| 4SNPs + 7Clinical | 0.912 (0.894-0.920) | 0.920 | 0.786 | 0.901 | 0.964 |
| 2SNPs + 7Clinical | 0.906 (0.887-0.916) | 0.886 | 0.786 | 0.872 | 0.963 |
| 4SNPs | 0.664 (0.638-0.674) | 0.874 | 0.429 | 0.813 | 0.905 |
| LG model |  |  |  |  |  |
| 4SNPs + 7Clinical | 0.912 (0.899-0.912) | 0.914 | 0.786 | 0.897 | 0.964 |
| 2SNPs + 7Clinical | 0.908 (0.893-0.914) | 0.886 | 0.786 | 0.872 | 0.963 |
| 4SNPs | 0.664 (0.652-0.674) | 0.874 | 0.429 | 0.813 | 0.905 |
| SVC model |  |  |  |  |  |
| 4SNPs + 7Clinical | 0.914 (0.901-0.918) | 0.834 | 0.893 | 0.842 | 0.980 |
| 2SNPs + 7Clinical | 0.906 (0.894-0.911) | 0.800 | 0.893 | 0.813 | 0.979 |
| 4SNPs | 0.667 (0.660-0.670) | 0.874 | 0.429 | 0.813 | 0.905 |
| Neural network multi-layer perceptron classifier |  |  |  |  |  |
| 4SNPs + 7Clinical | 0.904 (0.897-0.908) | 0.897 | 0.821 | 0.887 | 0.969 |
| Random forest |  |  |  |  |  |
| 4SNPs + 7Clinical | 0.871 (0.829-0.882) | 0.714 | 0.893 | 0.739 | 0.977 |
| Validation cohort |  |  |  |  |  |
| SGD model |  |  |  |  |  |
| 4SNPs + 7Clinical | 0.899 (0.887-0.904) | 0.810 | 0.926 | 0.829 | 0.983 |
| 4SNPs | 0.542 (0.526-0.545) | 0.930 | 0.132 | 0.802 | 0.849 |

AUC, area under the receiver operating characteristic curve; PRC, precision recall curve; 95%CI, 95% confidence interval; SGD, Stochastic gradient descent Classifier; LG, Logistic Regression; SVC, Linear Support Vector Classifier; 7 clinical features are age, hypertension, nephrolithiasis, glucose, serum urate, blood urea nitrogen and serum creatinine; 4 SNP variations were rs3775948.GG of *SLC2A9/GLUT9*, rs504915.AA of *SLC22A12/URAT1*, rs2231142.GG of *ABCG2*, and rs11231463.GG of *SLC22A9/OAT7*; 2 SNP variations were rs3775948.GG of *SLC2A9/GLUT9*, rs504915.AA of *SLC22A12/URAT1*; 4SNP-LASSO were rs11227805.TT, rs3775948.GG, rs504915.AA and rs7679724.TT.

**Supplementary Table S3 Performances of models under XGBoost for RUE in the internal test sets**

|  | AUC (95%CI) | Sensitivity | Specificity | Accuracy | PRC |
| --- | --- | --- | --- | --- | --- |
| SGD model | 0.895 (0.878-0.903) | 0.817 | 0.857 | 0.823 | 0.973 |
| LG model | 0.893 (0.879-0.900) | 0.783 | 0.893 | 0.798 | 0.979 |
| SVC model | 0.889 (0.877-0.897) | 0.829 | 0.821 | 0.828 | 0.967 |
| Neural network multi-layer perceptron classifier | 0.869 (0.857-0.876) | 0.771 | 0.857 | 0.783 | 0.971 |
| Random forest | 0.864 (0.807-0.875) | 0.771 | 0.786 | 0.773 | 0.957 |

18 variables under XGBoost: serum urate、glucose、eGFR、creatinine clearance rate、blood urea nitrogen、body mass index、low-density lipoprotein、diastolic blood pressure、serum creatinine、age、nephrolithiasis、history of smoking、systolic blood pressure、hypertension、rs7679724.TT、rs3775948.GG、rs57633992.AC、rs2762353.GG)
